# Transcriptome analysis of primary monocytes from HIV-positive patients with differential responses to antiretroviral therapy

**DOI:** 10.1186/1743-422X-10-361

**Published:** 2013-12-27

**Authors:** Jing Qin Wu, Tara Ruth Sassé, Monica Miranda Saksena, Nitin K Saksena

**Affiliations:** 1 School of Biomedical Sciences and Pharmacy, Faculty of Health, The University of Newcastle, University Drive, Callaghan, Newcastle, NSW, 2308, Australia; 2 Retroviral Genetics Division, Center for Virus Research, Westmead Millennium Institute & Westmead Hospital, University of Sydney, Westmead, Sydney, NSW, 2145, Australia; 3 Herpes Virus Pathogenesis Lab, Center for Virus Research, Westmead Millennium Institute, University of Sydney, Westmead, Sydney, NSW, 2145, Australia

**Keywords:** HIV, Monocyte, Transcriptome, HAART, Virological failure, Antigen presentation

## Abstract

**Background:**

Despite the significant contributions of monocytes to HIV persistence, the HIV-monocyte interaction remains elusive. For patients on antiretroviral therapy, previous studies observed a virological suppression rate of >70% and suggested complete viral suppression as the primary goal. Although some studies have reported genetic dysregulations associated with HIV disease progression, research on *ex vivo*-derived monocytic transcriptomes from HIV+ patients with differential responses to therapy is limited. This study investigated the monocytic transcriptome distinctions between patients with sustained virus suppression and those with virological failure during highly active antiretroviral therapy (HAART).

**Methods:**

Genome-wide transcriptomes of primary monocytes from five HIV+ patients on HAART who sustainably controlled HIV to below detection level (BDL), five HIV+ patients on HAART who consecutively experienced viremia, and four healthy HIV sero-negative controls were analyzed using Illumina microarray. Pairwise comparisons were performed to identify differentially expressed genes followed by quantitative PCR validation. Gene set enrichment analysis was used to check the consistency of our dataset with previous studies, as well as to detect the global dysregulations of the biological pathways in monocytes between viremic patients and BDLs.

**Results:**

Pairwise comparisons including viremic patients versus controls, BDL versus controls, and viremic patients versus BDLs identified 473, 76, and 59 differentially expressed genes (fold change > 2 and FDR < 0.05), respectively. The reliability of our dataset was confirmed by gene set enrichment analysis showing that 6 out of 10 published gene lists were significantly enriched (FDR < 0.01) in at least one of the three pairwise comparisons. In the comparison of viremic patients versus BDLs, gene set enrichment analysis revealed that the pathways characterizing the primary functions of monocytes including antigen processing and presentation, FcγR mediated phagocytosis, and chemokine signaling were significantly up-regulated in viremic patients.

**Conclusions:**

This study revealed the first transcriptome distinctions in monocytes between viremic patients and BDLs on HAART. Our results reflected the outcome balanced between the subversion of the monocyte transcriptome by HIV and the compensatory effect adapted by host cells. The up-regulation of antigen presentation pathway in viremic patients particularly highlighted the role of the interface between innate and adaptive immunity in HIV disease progression.

## Introduction

Monocytes, a key cell type in innate and adaptive immunity, have a propensity to differentiate into macrophages or dendritic cells [1,2]. This differentiation ability, along with the activities of antigen presentation, migration, chemotaxis, and phagocytosis [3], enables them to play crucial roles in HIV pathogenesis. Though less permissive to HIV infection than T cells and macrophages [4,5], monocytes could be infected by HIV [6,7] and infectious virus can be isolated from circulating monocytes in untreated patients and highly active antiretroviral therapy (HAART) responders [4,8]. By harboring and trafficking HIV into various tissue compartments through differentiating into tissue macrophages or dendritic cells, monocytes serve as important viral reservoirs [9,10]. During therapy, monocytes can maintain HIV replication throughout HAART as antiretroviral drugs may not block viral replication in monocytes as efficiently as they do in CD4+ T cells [10]. Moreover, undifferentiated monocytic precursor cells (such as CD34+ progenitor cells) infected by HIV may pass the virus to progeny monocytes and keep on renewing the viral pool in peripheral blood monocytes [11,12].

Despite the significant contributions of monocytes to HIV persistence, the underlying pathogenic mechanism is not fully understood. To better understand HIV pathogenesis at the genomic level, genome-wide transcriptomic studies of monocytes and monocyte-derived macrophages (MDM) have been carried out. For example, studies using monocytes/MDM infected by HIV *in vitro* revealed the key areas of monocyte dysfunctions related to inflammation [13], cytokine networks [14], cell cycle [15], cytoskeleton [16], and signaling pathways [17,18]. Other studies using *ex vivo*-derived monocytes identified an anti-apoptosis gene signature in viremic patients [19], a mixed phenotype with both increased and decreased pro-inflammatory features in patients with high viral load [20,21], and a novel candidate gene NAMPT correlating with the viral load in therapy-naïve patients [22]. Recently, by comparing the monocyte transcriptomes from HIV+ progressors and therapy naïve non-progressors, we have shown the systematic alteration of the interrelated pathways such as Toll-like receptor (TLR) signaling and cytokine-cytokine receptor interaction in viremic patients [23]. Although these studies have provided large datasets to facilitate our understanding, current knowledge on the dysregulations of monocytic transcriptome during HIV disease progression remains far from complete. In particular, none of the previous studies has looked into the global dysregulations of the biological pathways in monocytes from patients with sustained virus suppression versus those with virological failure during HAART, as we previously did on T cell subsets [24]. Regarding the virological suppression rate by HAART, the study on the patients receiving HAART for 12 months in Nigeria has observed a virological suppression rate of 76.7% versus a virological failure rate of 23.4% [25], whereas another study on the UK cohort has reported that 73.5% of the patients initiating HAART achieved complete virological suppression within 6 months [26].

In order to get a better insight into the dysregulations of monocytic transcriptomes from HIV+ patients with differential responses to antiretroviral therapy, this study analyzed transcriptomes of primary circulating monocytes from HIV+ patients on HAART who sustainably controlled HIV to below detection level (BDL), HIV+ patients on HAART who consecutively experienced viremia (VIR), and 4 sero-negative controls (CTR; Table 1) using Illumina HumanHT-12 v3 Expression BeadChip. Our analysis at the gene set level has shown that in the comparison of VIR versus BDL, the pathways characterizing the primary functions of monocytes including antigen processing and presentation, FcγR mediated phagocytosis, and chemokine signaling were significantly up-regulated in the VIR group. These results reflected the outcome balanced between the subversion of monocyte transcriptome by HIV and the compensatory effect adapted by host cells during disease progression following therapy. In particular, the up-regulation of antigen presentation pathway in the VIR group highlighted the role of the interface between innate and adaptive immunity in HIV disease progression.

**Table 1:** Clinical profiles of study patients

**Patient**	**Group**	**Age**	**Gender**	**Viral load (copies/ml)**	**CD4 (cells/μl)**	**CD8 (cells/μl)**	**Total months on HAART**	**RNA integrity number**
VIR1	VIR	54	F	87	418	473	14.8	10
VIR2	VIR	52	M	787	286	916	19.7	9.8
VIR3	VIR	31	F	194	300	548	23.2	9.9
VIR4	VIR	49	M	177	674	1131	14.3	10
VIR5	VIR	33	M	169	100	861	15.8	10
BDL1	BDL	45	M	< 40	796	1958	18.5	10
BDL2	BDL	48	F	< 40	459	1250	14.2	10
BDL3	BDL	43	F	< 40	427	569	24.0	10
BDL4	BDL	57	M	< 40	384	1728	14.3	10
BDL5	BDL	37	M	< 40	241	587	16.9	9.9

VIR1-5: patients on HAART consecutively experiencing viremia (viral load <1,000); BDL1-5: patients on HAART with sustained below detection level of plasma viral load (viral load < 40 copies of HIV RNA/ml).

## Results

### Cluster analysis and identification of differentially expressed genes

**Figure 1:**
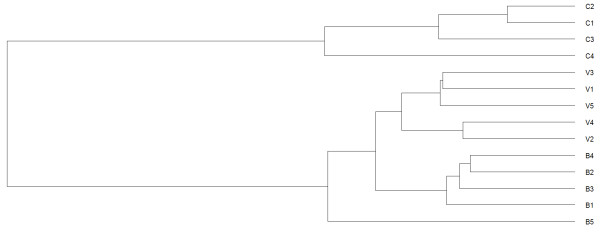
**Clustering analysis of gene expression profiles of monocytes.** The label V represents for viremic patients, B for BDLs, and C for controls.

Genome-wide transcriptomes of primary monocytes from 5 HIV+ patients on HAART who consecutively experienced viremia, 5 HIV+ patients on HAART who sustainably controlled HIV to below detection level, and 4 healthy HIV sero-negative controls (5 VIR, 5 BDL, and 4 CTR; Table 1) were analyzed using Illumina microarray. The hierarchical clustering analysis revealed that the VIR group formed an independent cluster from the BDL group and these HIV+ groups further combined into a distinct cluster from the CTR group (Figure 1). Pairwise comparisons between the three groups were carried out and differentially expressed genes (DEGs) with FDR < 0.05 and fold change > 2 were identified for each comparison. For the comparison of VIR versus CTR, 473 DEGs were identified (324 up-regulated and 149 down-regulated; Additional file 1). For the comparison of BDL versus CTR, 76 DEGs were found (45 up-regulated and 31 down-regulated; Additional file 1). When the VIR group was compared to the BDL group, 59 DEGs were detected (48 up-regulated and 11 down-regulated; Table 2). These 59 DEGs were uploaded to DAVID (Database for Annotation, Visualization, and Integrated Discovery) for the detection of DEGs overlapping with the genes in HIV interaction database [23]. Fourteen DEGs were present in HIV interaction database, covering 24% of our list (Table 2), which gave the initial confirmation of the reliability of our dataset at the discrete gene level (Additional file 2).

**Table 2:** Differentially expressed genes in the comparison of VIR versus BDL

**ProbeID**	**Gene symbol**	**Entrez gene name**	**logFC**	**FDR**
4540239	*DEFA1*	Defensin, alpha 1	-2.06	0.018
2970747	*DEFA3*	Defensin, alpha 3	-1.74	0.012
7150170	LOC728358	NA	-1.54	0.020
50088	HCFC1R1	Host cell factor C1 regulator 1 (XPO1 dependent)	-1.47	0.003
2750647	C20orf27	Chromosome 20 open reading frame 27	-1.17	0.005
3390068	E4F1	E4F transcription factor 1	-1.09	0.003
4280661	TMUB2	Transmembrane and ubiquitin-like domain containing 2	-1.08	0.003
2490333	ZNF467	Zinc finger protein 467	-1.06	0.036
1820053	SLC43A2	Solute carrier family 43, member 2	-1.05	0.010
6940647	C16orf58	Chromosome 16 open reading frame 58	-1.01	0.034
1570255	DEF6	Differentially expressed in FDCP 6 homolog (mouse)	-1.00	0.017
2630017	*TPST2*	Tyrosylprotein sulfotransferase 2	1.00	0.027
5810008	DLGAP4	Discs, large (Drosophila) homolog-associated protein 4	1.01	0.017
4050475	KIAA0319L	KIAA0319-like	1.01	0.048
730632	LSP1	Lymphocyte-specific protein 1	1.02	0.017
7160711	MAP2K3	Mitogen-activated protein kinase kinase 3	1.02	0.020
4200025	OAF	OAF homolog (Drosophila)	1.02	0.008
1510414	MGRN1	Mahogunin ring finger 1, E3 ubiquitin protein ligase	1.04	0.017
2490072	PRAM1	PML-RARA regulated adaptor molecule 1	1.04	0.020
7320494	ZFPM1	Zinc finger protein, FOG family member 1	1.04	0.003
6450092	*C5AR1*	Complement component 5a receptor 1	1.05	0.031
7570068	*EP300*	E1A binding protein p300	1.05	0.044
770168	CLPTM1	Cleft lip and palate associated transmembrane protein 1	1.06	0.005
1070044	GMIP	GEM interacting protein	1.06	0.017
7400053	SLC22A18	Solute carrier family 22, member 18	1.06	0.041
3800307	SHKBP1	SH3KBP1 binding protein 1	1.07	0.005
830750	NCF1B	Neutrophil cytosolic factor 1B pseudogene	1.07	0.012
7050484	ZYX	Zyxin	1.08	0.008
4640064	LTBR	Lymphotoxin beta receptor (TNFR superfamily, member 3)	1.08	0.008
2000224	FAM89B	Family with sequence similarity 89, member B	1.09	0.018
1940021	*GRN*	Granulin	1.09	0.017
2480288	GPAA1	Glycosylphosphatidylinositol anchor attachment 1	1.09	0.022
2970356	ALDH3B1	Aldehyde dehydrogenase 3 family, member B1	1.13	0.005
5570767	*PPP2R1A*	Protein phosphatase 2, regulatory subunit A, alpha	1.13	0.006
5890072	TRAPPC9	Trafficking protein particle complex 9	1.14	0.001
6960026	STXBP2	Syntaxin binding protein 2	1.14	0.000
5910019	*C1QB*	Complement component 1, q subcomponent, B chain	1.17	0.019
270437	*ACTN4*	Actinin, alpha 4	1.18	0.000
5570242	PFKL	Phosphofructokinase, liver	1.20	0.004
840669	LOC654346	NA	1.22	0.017
4920546	ARSA	Arylsulfatase A	1.24	0.001
2650092	JOSD2	Josephin domain containing 2	1.25	0.000
4760255	*ARHGDIA*	Rho GDP dissociation inhibitor (GDI) alpha	1.26	0.004
6480333	TCIRG1	T-cell, immune regulator 1, ATPase, H + transporting, lysosomal V0 subunit A3	1.29	0.001
4180494	*ITGAL*	Integrin, alpha L (antigen CD11A (p180), lymphocyte function-associated antigen 1; alpha polypeptide)	1.30	0.010
6620609	ABTB1	Ankyrin repeat and BTB (POZ) domain containing 1	1.30	0.021
150154	FAM108A3	Family with sequence similarity 108, member A3, pseudogene	1.30	0.000
4730349	PNKD	Paroxysmal nonkinesigenic dyskinesia	1.31	0.040
2140392	ZNF580	Zinc finger protein 580	1.32	0.006
4890068	*MAP3K11*	Mitogen-activated protein kinase kinase kinase 11	1.32	0.001
7550500	JUNB	Jun B proto-oncogene	1.39	0.028
7650128	ECGF1	Thymidine phosphorylase	1.40	0.012
4220603	SPI1	Spleen focus forming virus (SFFV) proviral integration oncogene spi1	1.43	0.005
5090632	LGALS9	Lectin, galactoside-binding, soluble, 9	1.45	0.006
20673	*BSG*	Basigin (Ok blood group)	1.61	0.000
770754	TTYH3	Tweety homolog 3 (Drosophila)	1.72	0.001
6580059	UCP2	Uncoupling protein 2 (mitochondrial, proton carrier)	1.79	0.023
130403	RNF19B	Ring finger protein 19B	1.83	0.004
6370315	*HLA-DRB5*	Major histocompatibility complex, class II, DR beta 5	5.66	0.012

The differentially expressed genes were sorted by fold change.

Gene symbols in italic indicated genes in HIV interaction database.

*FDR*: false discovery rate; *NA*: not available.

### qPCR validation and the comparison to previous microarray studies

To further confirm the DEGs from microarray analysis, mRNA expression levels of the selected DEGs were measured by quantitative PCR (qPCR; Table 3). The DEGs were selected based on the coverage of different levels and directions of fold change, different ranges of FDR values, and/or biological significance. The cohort for qPCR validation consisted of 10 viremic patients, 10 BDLs, and 9 healthy controls (including all the original samples used in the microarray). The fold changes for each pairwise comparison evaluated by qPCR were fully consistent with the results obtained from microarray, which confirmed the reliability of our microarray data.

**Table 3:** qPCR confirmation of differentially expressed genes

**Gene**	**Comparison**	**qPCR**	**Microarray**	**Gene**	**Comparison**	**qPCR**	**Microarray**
ACTN4	VIRvsCTR	3.1	3.6	IL1B	VIRvsCTR	-12.5	-13.2
ACTN4	VIRvsBDL	1.8	2.3	IL1B	BDLvsCTR	-17.0	-20
ITGAL	VIRvsCTR	1.9	2.4	CSK	VIRvsCTR	3.7	2.9
ITGAL	VIRvsBDL	2.3	2.5	CFL1	VIRvsCTR	2.5	2.2
GNAI2	VIRvsCTR	2.7	2.9	CD37	VIRvsCTR	2.3	2.4
SERPING1	VIRvsCTR	3.8	2.6	C1QB	VIRvsBDL	2.6	2.3
IL8	VIRvsCTR	-4.6	-5.0	C1QB	VIRvsCTR	4.1	4.3

Fold change by qPCR was obtained from the mean expressions of the tested genes in each group. The cohort for qPCR validation consisted of 10 viremic patients, 10 BDLs, and 9 healthy controls (including all the original samples used in the microarray). All values represent fold changes between expression levels of the first group versus expression levels of the second group. Minus sign indicates down-regulation in the first group whereas positive sign indicates up-regulation in the first group. Housekeeping gene GAPDH was used as an internal control and the normalizer in qPCR.

We then compared our dataset with published DEG lists derived from the studies on monocyte/MDM transcriptomes modulated by HIV since 2002 [15,16,19,21,22,27-30]. The GSEA showed that in our transcriptome dataset, 6 out of the 10 published gene lists were significantly enriched (FDR < 0.01, the most stringent cut off) in at least one of the 3 pairwise comparisons, whereas the remaining 4 gene lists reached the relaxed significance level (FDR < 0.25) in all of the 3 pairwise comparisons (Table 4). Nevertheless, the highly significant enrichment of the majority of these gene lists (FDR < 0.01) demonstrated general consistency of our data with previous studies.

**Table 4:** **The GSEA of our dataset compared with the published gene lists derived from monocyte**/**MDM transcriptomes modulated by HIV**

**Gene set name**	**Pathways/biological functions DEGs involved in HIV infection/disease progression**	**Study description**	**Reference**	**Gene set size**	**VIRvs BDL FDR**	**VIRvs CTR FDR**	**BDLvs CTR FDR**
Cicala_Cytokine _Chemokine	Chemokine and cytokine	*in vitro* gp120-treated vs mock-treated MDM	[16]	34	0.288	0.000***	0.000***
Coberley_HIV_ Induced_Repressed	Cell cycle regulators, translation, cell signaling, TNF, MAPK	*in vitro* HIV-infected vs mock-treated MDM	[15]	38	0.07*	0.192*	0.226*
Woelk_InteferonStimuated	Interferon stimulated genes, host defense genes	*in vitro* HIV-infected vs mock-treated MDM	[30]	12	0.004***	0.000***	0.007***
Vazquez_HIVinduced _MDM	Signal transduction, transcription, cell cycle and apoptosis, adhesion molecules and receptors, chemokines and cytokines, proteases and protease inhibitors, metabolism	*in vitro* HIV-infected vs mock-treated MDM	[28]	124	0.059*	0.096*	0.159*
Wen_HIVvsMock_U937	Signaling components, transcription factors, cytokines, apoptotic and anti-apoptotic factors, growth factors, anti-HIV infection genes	*in vitro* HIV-infected vs mock-infected U937 human promonocytes	[29]	33	0.094*	0.008***	0.004***
TILTON_CytokineLevel _Correlation	Type I interferon responses, NF-κB, mitogen-activated protein kinase, Jun signaling pathways, general immune activation, immune down-regulation, protein degradation, protein secretion, and apoptosis	*ex vivo* correlations between changes in gene expression values and changes in monocyte cytokine levels in HIV+ patients on and off therapy	[21]	1295	0.222*	0.161*	0.156*
Giri_Apoptosis _StableDifferential	Apoptosis-related gene signatures, TNF-α signaling, CD40L/CD40 signaling, MAPK signaling, p53 modulation	*ex vivo* HIV+ patients vs healthy controls	[19]	36	0.006***	0.177*	0.003***
Van_HIV_Serostatus _associated _validated	Apoptosis, cell cycle, transcriptional regulation, immune response, protein trafficking, lipid metabolism	*ex vivo* HIV+ patients vs healthy controls	[22]	24	0.018**	0.003***	0.000***
Gekonge_Overlap_ ControlStimulatedvsControl_HIVvsControl	TLR2-agonist stimulated gene signature TNF (NFκB), p53 and MAPK networks	*ex vivo* and *in vitro* HIV+ patients vs healthy controls stimulated vs non-stimulated controls	[27]	62	0.229*	0.008***	0.015**
Gekonge_HIVvsControl	ERK/MAPK, TNF/IL6 (NFκB) and p53 gene networks, apoptosis-related gene signatures	*ex vivo* HIV+ patients vs healthy controls	[27]	281	0.138*	0.066*	0.061*

*DEGs*: differentially expressed genes; gene set size: number of genes in the published list; *FDR*: false discovery rate; *VIR*: the viremic patients; *CTR*: the healthy control group; *MDM*: monocyte-derived macrophages; vs: versus. *FDR < 0.25 (default cutoff); **FDR < 0.05(more stringent); ***FDR < 0.01(most stringent).

### Pathway analysis by GSEA

As we aimed to identify monocyte dysfunction in relation to HIV disease progression, our subsequent pathway analysis focused on the comparison between VIR and BDL groups. GSEA was attempted using the KEGG pathways including 186 gene sets. Unlike DEG approach, GSEA uses the whole gene expression dataset to identify enriched pathways and is better able to detect small coordinated changes in gene expression in the context of gene set [31,32].

In the comparison of VIR versus BDL, 26 pathways were significantly up-regulated in the VIR group (FDR < 0.05/stringent cut off; Additional file 3), whereas no pathway down-regulated in the VIR group passed this threshold. These 26 significantly up-regulated pathways can be grouped into 3 functional categories including immune-related pathways (n = 10), disease (n = 10), and metabolism pathways (n = 6; Table 5; Additional file 3). We chose to focus on the immune-related pathways as they have the most direct relevance to the immune dysfunction of monocytes.

**Table 5:** **The ten immune**-**related pathways significantly up**-**regulated in viremic patients versus BDLs**

**Gene set name**	**GS size**	**NOM p-val**	**FDR**	**Core enrichment genes contributing to the pathway enrichment**
KEGG_INTESTINAL_IMMUNE_ NETWORK_FOR_IGA_PRODUCTION	48	0.00	0.014	HLA-DRB5, HLA-DRB1, LTBR, ITGB7, CD40, TNFSF13, HLA-DOA, CCR9
KEGG_ANTIGEN_PROCESSING_ AND_PRESENTATION	88	0.00	0.016	HLA-DRB5, HLA-DRB1, CD4, HLA-C, IFI30, NFYC, TAPBP, HSPA1B, CD74, HLA-DOA, TAP2, HLA-E, PDIA3, PSME1, HSP90AB1, RFX5, CALR, KIR2DS5, HLA-F
KEGG_ADHERENS_JUNCTION	73	0.00	0.016	ACTN4, EP300, TCF7L2, PTPN6, IQGAP1, CSNK2B, SRC, FYN, RAC1, CSNK2A2, WASF2, PVRL4, PTPN1, BAIAP2, CTNNB1, PVRL2, ACTB
KEGG_HEMATOPOIETIC_CELL_ LINEAGE	87	0.00	0.016	HLA-DRB5, HLA-DRB1, FLT3LG, CD4, TNF, CD37, IL1B, CD9, CD36, EPOR, ANPEP, CD1A, CD19, ITGA2B, FCER2, CD1D, FCGR1A
KEGG_CELL_ADHESION_ MOLECULES_CAMS	134	0.00	0.017	HLA-DRB5, HLA-DRB1, ITGAL, ITGB7, CD40, CD4, HLA-C, SELL, PECAM1, SPN, HLA-DOA, ICAM2, HLA-E, ICAM3, CLDN3, PVRL2, JAM3, HLA-F, CD28, CLDN18, PVR, CD99, ICOS, PDCD1LG2, CD80, CLDN5, NEO1, PDCD1, ITGAM, PTPRF, HLA-DRA, CNTN2, HLA-B, CD58, NLGN3, CTLA4, ITGA4, SIGLEC1
KEGG_LYSOSOME	119	0.00	0.023	ARSA, ACP2, SGSH, CTSZ, MCOLN1, CTSA, CTSD, SORT1, FUCA1, MAN2B1, GBA, TCIRG1, NAPSA, IDUA, NAGPA, M6PR, ATP6V0D1, NEU1, ACP5, GGA1, LAPTM5, CD63, AP1S1, AP3B1, AP3D1, NAGA, CLN3, IDS, TPP1
KEGG_NEUROTROPHIN_ SIGNALING_PATHWAY	125	0.00	0.025	YWHAE, ARHGDIA, RPS6KA4, SH2B2, MAP2K2, MAPKAPK2, MAPK7, SORT1, CSK, RELA, SHC1, PIK3CG, KIDINS220, CRK, IRAK1, BAX, SH2B3, RAC1, MAP2K5, AKT1, CALM1, MAP3K3, MAPK9, ABL1, RPS6KA3, SH2B1, IRS1, RAF1, HRAS, MAPK14
KEGG_FC_GAMMA_R_MEDIATED_PHAGOCYTOSIS	92	0.00	0.029	MARCKSL1, NCF1, PIP5K1C, VASP, HCK, PIK3CG, DNM2, CRK, SYK, ARPC4, ARPC1A, SPHK1, RAC1, ARPC5L, LYN, AKT1, WASF2, VAV1, ARPC1B, LOC653888, FCGR1A, RAF1, PRKCA, GSN, DNM1, PAK1, VAV2, CFL1, AKT2
KEGG_CHEMOKINE_SIGNALING_ PATHWAY	185	0.00	0.038	PLCB2, NCF1, GNB2, ELMO1, CX3CR1, GRK6, PPBP, GNAI2, CSK, GNB4, RELA, SHC1, HCK, ARRB1, PIK3CG, CRK, RASGRP2, CCR1, GNGT2, GNG5, GNG3, STAT5B, RAC1, LYN, AKT1, CCL2, JAK2, VAV1, GNG8, CCR2, CCR9, PRKACA, CCR7, XCR1, TIAM2, GNG12, RAF1, ARRB2, HRAS, CXCR5, PTK2B, PAK1, CCR3, VAV2
KEGG_COMPLEMENT_AND_ COAGULATION_CASCADES	68	0.01	0.038	C1QB, C1QC, C5AR1, SERPING1, C1QA, C2, KLKB1, C3AR1, THBD, SERPINA1, C4A, SERPINA5

*GS* size: Gene set size (number of genes in a particular gene set); *NOM p*-val: nominal p value; *FDR*: false discovery rate.

Out of the ten immune-related pathways (Table 5), two were involved in cell differentiation and development (hematopoietic cell lineage and neurotrophin signaling), two were associated with transendothelial migration (cell adhesion molecules and adherens junctions), and the remaining six covered all the major aspects of innate immunity, including chemokine signaling, IgA production, complement cascade, phagocytosis, lysosome, and antigen presentation. All the core enrichment genes contributing to the up-regulation of the immune-related pathways are listed in Table 5. Since the pathways of antigen presentation, phagocytosis, and chemokine signaling characterize the primary functions of monocytes, we further inspected their core enrichment genes.

### Up-regulation of antigen presentation pathway in VIR versus BDL

**Figure 2:**
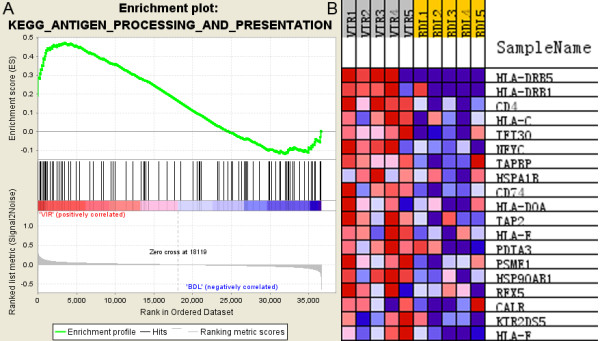
**Enrichment plot and heat map for the gene set of antigen processing and presentation pathway by GSEA. A.** Enrichment plot for monocytes from the VIR group (VIR versus BDL). **Bottom**, plot of the ranked list of all genes. Y axis, value of the ranking metric; X axis, the rank for all genes. Genes whose expression levels are most closely associated with the VIR or BDL group get the highest metric scores with positive or negative sign, and are located at the left or right edge of the list. **Middle**, the location of genes from the antigen processing and presentation pathway within the ranked list. **Top**, the running enrichment score for the gene set as the analysis walks along the ranked list. The score at the peak of the plot is the enrichment score (ES) for this gene set and those genes appear before or at the peak are defined as core enrichment genes for this gene set. **B.** Heat map of the core enrichment genes corresponding to **A.** The genes that contribute most to the ES, i.e., genes that appear in the ranked list before or at the peak point of ES, are defined as core enrichment genes. Rows, genes; columns, samples. Range of colors (red to blue) shows the range of expression values (high to low).

**Figure 3:**
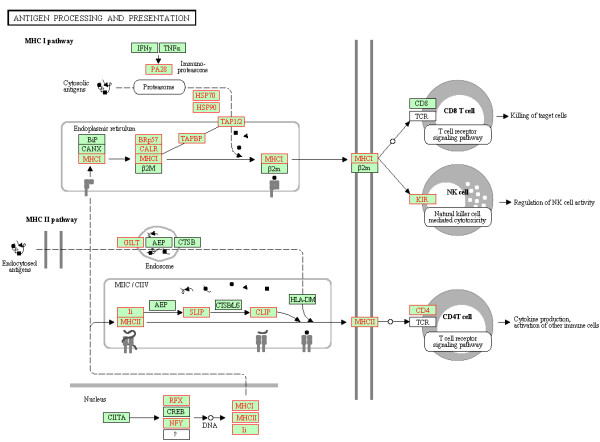
**Coordinately up**-**regulated genes of antigen processing and presentation pathway in monocytes from the VIR group (VIR versus BDL).** The pathway figure is adapted from Kyoto Encyclopedia of Genes and Genomes (KEGG; http://www.genome.jp/kegg/). The proteins encoded by the coordinately up-regulated genes in the VIR group are highlighted in red.

The antigen processing and presentation pathway (HSA04612) was significantly up-regulated in the VIR group compared to the BDL group (FDR = 0.016). The enrichment plot and the heat map of the core enrichment genes for antigen presentation pathway are displayed as representatives to illustrate the GSEA output in Figure 2. Figure 2B shows not only the coordinated up-regulation of these core enrichment genes in the VIR group as a combination from all the viremic patients, but also the variations in gene expression between subjects within each group. For example, while VIR2 had lower expression of the core enrichment genes than VIR1, VIR2 still exhibited higher expression than the majority of the patients from the BDL group. Nineteen out of 88 gene members of this pathway were core enrichment genes (Table 5; Figure 3), which included 6 MHC molecules (MHC I: HLA-C, HLA-E, and HLA-F; MHC II: HLA-DOA, HLA-DRB1, and HLA-DRB5); 2 transcription factors associated with MHC transcription (NFYC and RFX5), 4 molecules related to antigen digestion (MHC I: PSME1/PA28 alpha, HSP90AB1, HSPA1B/HSP70; MHC II: IFI30/GILT), 5 molecules involved in antigen transport and loading (MHC I: TAP2 and TAPBP, CALR, and PDIA3/BRp57; MHC II: CD74/CLIP), and 2 cell surface molecules (CD4 and KIR2DS5).

### Up-regulation of phagocytosis pathway in VIR versus BDL

**Figure 4:**
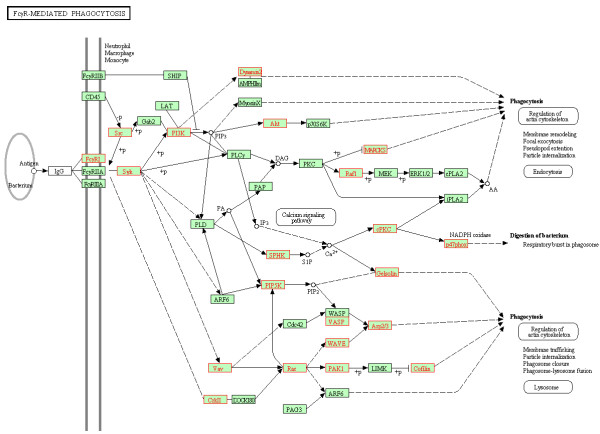
**Coordinately up**-**regulated genes of FcγR mediated phagocytosis pathway in monocytes from the VIR group (VIR versus BDL).** The pathway figure is adapted from Kyoto Encyclopedia of Genes and Genomes (KEGG; http://www.genome.jp/kegg/). The proteins encoded by the coordinately up-regulated genes in the VIR group are highlighted in red.

FcγR-mediated phagocytosis (HSA04666) was significantly up-regulated in the VIR group versus the BDL group (FDR = 0.029). It was exemplified by the increased Fc receptor expression (FCGR1A), the up-regulation of SRC kinases (LYN and HCK) coupled with receptor binding, and the subsequently triggered kinases activating a range of downstream effectors (Table 5). Figure 4 shows that the majority of these 29 core enrichment genes (92 genes in total) spread along the arms of SYK - PI3K - AKT/Dynamin, SPHK - PRKCA (cPKC) - SPHK1 (NADPH oxidase involved in microorganism digestion), PIP5K - VASP/WASP - ARP2/3 (actin related protein), and VAV/CRKII – RAC - PAK1 - CFL1 (Cofilin, an actin-modulating protein). The end effectors were involved in both cytoskeleton rearrangement crucial for phagosome formation (ARP2/3, MARCKS, Gelsolin, Cofilin, and Dynamin) and the release of reactive oxygen species (p47phox) for microorganism degradation.

### Up-regulation of chemokine signaling pathway in VIR versus BDL

**Figure 5:**
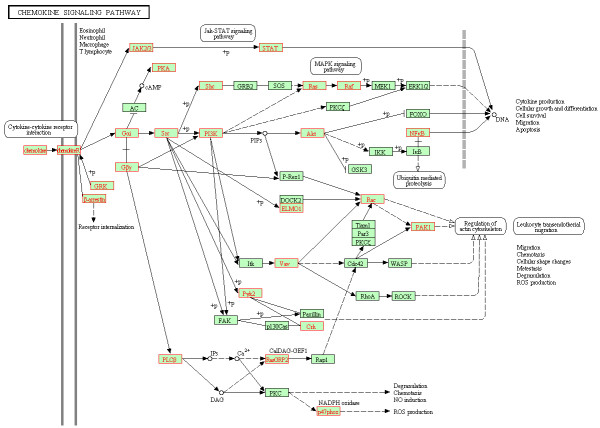
**Coordinately up**-**regulated genes of chemokine signaling pathway in monocytes from the VIR group (VIR versus BDL).** The pathway figure is adapted from Kyoto Encyclopedia of Genes and Genomes (KEGG; http://www.genome.jp/kegg/). The proteins encoded by the coordinately up-regulated genes in the VIR group are highlighted in red.

Forty-four out of 185 members of chemokine signaling pathway (HSA04062) were coordinately up-regulated (core enrichment genes) in the VIR group versus the BDL group (FDR = 0.038; Table 5; Figure 5). Nine genes were involved in receptor interaction including 8 chemokine receptors (CCR1, CCR2, CCR3, CCR7, CCR9, CX3CR1, CXCR5, and XCR1) and 1 chemokine ligand (CCL2). Three genes were associated with receptor deactivation (GRK, ARRB1 and ARRB2/β-arrestin) and 8 were G proteins (GNAI2, GNB2, GNB4, GNG12, GNG3, GNG5, GNG8, and GNGT2), which initiated subsequent signaling cascades. The Gα subunit triggered signaling through three arms including SRC (SCK and LYN) – PI3K (PIK3CG) – AKT (AKT1) – NFκB (RELA), SHC (SHC1) – RAS (HRAS) – RAF (RAF1; MAPK pathway), and PYK2 – CRK. The Gβ subunit not only activated PLCβ (PLCB2) which led to reactive oxygen species production (p47phox), but also signaled through RAC1 and PAK1 resulting in regulation of actin cytoskeleton. The signaling arm of JAK2 – STAT5B was also up-regulated.

### Promoter motif analysis by GSEA

Promoter motif analysis by GSEA used gene sets that contained gene members sharing the same transcription factor binding site defined in the TRANSFAC database [33]. This analysis can identify the coordinated changes of the genes under the control of a certain transcriptional regulator. The GSEA revealed that 37 and 42 gene sets were significantly up- and down- regulated in the VIR group (FDR < 0.05) compared to the BDL group (Additional file 4). While the majority of the significantly altered gene sets contained regulatory motifs matching for the annotated transcription factors, 12 gene sets contained motifs which did not match any known transcription factor. The most significantly up- and down- regulated gene sets in the VIR group (both FDR = 0.03) had gene members sharing the regulatory motif for transcription factors NFκB and MYC, respectively.

## Discussion

Our study has provided the first snapshot into the transcriptome distinctions of the primary monocytes between HIV+ patients on HAART who consecutively experienced viremia and HIV+ patients on HAART who sustainably controlled HIV to below detection level (Table 1). The main objective of the study was to identify genomic signatures associated with HIV disease progression. The relevance of the identified DEGs was initially confirmed by querying HIV interaction database using DAVID (Table 2; Additional file 2). The comparison between our dataset and previous microarray studies on monocyte/MDM transcriptomes further confirmed the reliability of our data (Table 4). The previously identified gene sets, which were highly significant (FDR < 0.01) in our dataset, reflected the altered biological functions including cytokine networks, cell cycle, signaling pathways, metabolism, immune responses, and transcriptional regulation. These biological themes were also represented by the gene sets which only reached the relaxed significance threshold (FDR < 0.25). The failure of these gene sets to achieve higher statistical cut-offs could possibly be attributed to certain biological and/or technical variations across multiple microarray studies. For example, differences in the cohort such as age, gender, and ethnicity could contribute to the biological variations, whereas microarray inter-platform differences could contribute to the technical variations. Compared to DEG approach which focused on discrete genes, GSEA enabled a more comprehensive detection of genes contributing to the enrichment of the pathways correlated with disease progression. Therefore, the subsequent discussion focused on the pathways significantly associated with progressive phase of HIV disease revealed by GSEA. Since the immune-related pathways have the most direct relevance to HIV disease and this aspect has been under constant investigations, our subsequent discussions shall center on the immune-related pathways including antigen presentation, phagocytosis, and chemokine signaling.

At the transcriptome level, we observed an overall up-regulation of antigen processing and presentation pathway in the VIR group compared to the BDL group (FDR = 0.016; Figure 3; Table 5), which was manifested by the up-regulation of both endogenous (MHC-I) and exogenous (MHC-II) signaling branches. Five molecules from MHC-II pathway were detected as core enrichment genes including IFI30, CD74, HLA-DOA, HLA-DRB1, and HLA-DRB5 (Table 5). The coordinated up-regulation of HLA-DR was consistent with the previous studies reporting higher expression levels of surface HLA-DR on monocytes from HIV+ individuals compared to sero-negative controls [34-36]. As for the MHC-I pathway, the majority of the core enrichment genes were reported to have interactions with HIV, such as HSP90AB1 [37] and PA28 (PSME1) [38]. Moreover, 3 MHC-I molecules including HLA-C, HLA-E, and HLA-F were also detected as core enrichment genes with the coordinated up-regulation in the viremic patients. The importance of HLA-C was highlighted in a very recent study showing that increased HLA-C expression was correlated with increased likelihood of cytotoxic T lymphocyte (CTL) responses and frequency of viral escape mutation [39]. In addition, the substantially increased expression of MHC-I including HLA-C has been reported in monocytes in HIV progressors compared to HAART-suppressed patients, which was suggested to contribute to the generation of the dysfunctional naive CD8-low T cells that emerged during disease progression [40]. Taken together, our observation that the up-regulation of HLA-C expression on monocytes was associated with plasma viremia could possibly reflect a compensatory effect imposed by impaired CTL responses. However, this compensatory effect may be subverted by HIV, resulting in further CD8+ T cell dysfunction as proposed by Favre *et al*. [40].

As antigen presentation pathway is right at the interface between innate and adaptive immunity, its significant up-regulation in the viremic patients provides direct evidence that adaptive immune response is perturbed through the components of innate immunity during HIV disease progression. This interference is systematic throughout the process of antigen presentation as demonstrated by the core enrichment genes covering not only the aforementioned MHC molecules, but also the genes associated with antigen digestion, loading and transportation (Table 5). In fact, the dysregulation of antigen presentation pathway is not the only pathway altered at the interface. Other innate immunity pathways crucial for the development of adaptive immunity have also been associated with HIV disease progression by this and previous studies [23,24,41]. For example, this study detected that complement cascade regulating both B and T cell responses [42] was significantly up-regulated in the viremic patients (Table 5). Consistently, complement pathway was also found to be up-regulated in the viremic patients versus BDLs by our previous study on primary CD4+ T cells [24]. In addition, a recent study has reported that monocytes and complement system contributed to the tuberculosis-associated immune reconstitution inflammatory syndrome in HIV-TB co-infected patients [41]. Altogether, these studies have demonstrated the importance of complement components in HIV disease progression. Recently, another key component of innate immunity, Toll-like receptor signaling (TLR) pathway, has been found to be significantly down-regulated in monocytes from viremic patients versus long-term non-progressors [23]. The different direction of the changes may reflect the various aspects of HIV-host interactions that contribute to disease progression, such as HIV persistence and impairment of T cell functions. Despite this difference, all of the aforementioned studies point towards the adaptive immunity being perturbed at the interface where innate and adaptive immunity interact during HIV disease progression.

Consistent with the previous reports on phagocytosis dysfunction in monocytes upon HIV infection [43-45], we observed the significant up-regulation of FcγR-mediated phagocytosis pathway (HSA04666) in the VIR group versus the BDL group (FDR = 0.029; Figure 4). The expression of FCGR1A (FcγRI/CD64), the sole high affinity receptor for monomeric IgG, was coordinately increased along with other core enrichment genes in the viremic patients. This observation was consistent with the previous study reporting the increased expression of FcγRI on monocytes from acute HIV infection patients compared to chronically-infected individuals and healthy controls [46]. In addition, the previous study suggested that monocytes might expand and/or up-regulate the expression of this high affinity FcγR in response to the burst of viral replication. This host response may also account for the increased FCGR1A expression in the viremic patients on HAART, as detected in this study.

The perturbation of phagocytosis pathway was further manifested by the up-regulation of the genes encoding for kinases in early signaling events such as SRC kinases (HCK and LYN) and SYK, as well as the genes encoding for proteins involved in oxidative burst (SPHK1/NADPH oxidase) and cytoskeleton rearrangement (Table 5). The majority of the core enrichment genes, which spread along the signaling branches of PI3K – AKT, PIP5K – VASP/WASP/WAVE – ARP2/3, and VAV – RAC – PAK1 – CFL1, have also been implicated previously in HIV pathogenesis. A recent study using gene knockdown and pharmacological inhibition of SRC and AKT identified them as mediators of HIV-induced inhibition of autophagocytosis in bystander macrophages/monocytic cells [47]. The elevated AKT activity was also detected in HIV-infected macrophages, and PI3K/AKT inhibitors were suggested as a novel therapy for interfering with the establishment of HIV reservoirs [48]. In the signaling branch pointing towards ARP2/3, WAVE2 was involved in the activation of the actin polymerization nucleator ARP2/3 complex, which played a role in the migration of the viral core components toward the cell nucleus and the efficient infection of HIV in cell lines [49,50]. In addition, HIV Nef was found to activate the signaling branch of VAV – RAC – PAK (p21), which markedly enhanced the NADPH oxidase response [51]. Nef also mediated CFL1 phosphorylation, which was crucial for maintaining actin homeostasis [52]. Taken together, the up-regulated expression of the genes involved in various kinase cascades and the gene encoding for FcγRI may reflect the HIV interference with the host genetic machinery on the one hand, and the compensatory processes adapted by the host on the other hand during virological failure.

Chemokine signaling pathway (HSA04062) was also significantly up-regulated in the VIR group versus the BDL group (FDR = 0.038), which was manifested by the systematic up-regulation of the genes encoding for chemokine ligand 2 (CCL2/MCP-1) and chemokine receptors including CCR1, CCR2, CCR3, CCR7, CCR9, CX3CR1, CXCR5, and XCR1 (Table 5; Figure 5). The up-regulation of MCP-1 in viremic patients was also reported by Pulliam *et al*. [20], which was suggested to be involved in enhancing HIV production and spread [53]. In addition, it was shown that the sequential activation of SRC, MAPKs, and PI3K – AKT – NFκB pathways resulted in the increased expression of MCP-1 [54]. Given the fact that the majority of these genes overlapped with the core enrichment genes we detected, it is thus plausible to hypothesize that the up-regulation of MAPK and NFκB braches signaling through Gα subunit may be related to the coordinated up-regulation of MCP-1, which could contribute to HIV spread. This potential link could be one of the mechanisms underlying the association of the up-regulation of chemokine signaling pathway with virological failure during HAART.

In the comparison of VIR versus BDL, the promoter motif analysis yielded a list of significantly up- and down- regulated gene sets with members of each gene set containing the same binding site for a certain transcription factor (Additional file 4). The transcription factors implicated by these significant gene sets included some of the well-known regulators, such as SP (SP1 and SP3), NFκB (RELA binding motif), AP1, AP2, and CREB1, which have been demonstrated to play crucial roles in HIV transcription regulation [17]. Interestingly, the most significantly up-regulated gene set in the VIR group (FDR = 0.03) contained the binding motif for RELA, which was also identified as the core enrichment gene in chemokine signaling pathway in the VIR group. This overlap not only demonstrated the consistency between pathway and promoter motif analysis, but also implicated the complexity of the transcriptional regulation underlying HIV-monocyte interaction. In addition to the aforementioned transcription factors, some of the significant gene sets contained the binding motifs without matching any known transcription factor, which may suggest potential novel regulators that warrant future investigations.

A few limitations of this study should be noted. First, this study used a cross-sectional design, which could not provide dynamic findings from a longitudinal perspective. Secondly, this study used a relatively small sample size appropriate for the pilot investigation and future studies using larger sample size are thus warranted to further confirm the results. Finally, although the GSEA identified a panel of significantly altered gene sets with high relevance to HIV disease progression during therapy, the predictive nature of GSEA as the common limitation of statistical tools should be noted and interpretations should be made with caution. To overcome this limitation, future biological experiments should be conducted to directly confirm and further explore these findings.

## Conclusions

This study has revealed the first transcriptome distinctions in monocytes between HIV+ patients on HAART who consecutively experienced plasma viremia and HIV+ patients on HAART who sustainably controlled plasma viremia to below detection level. Compared to the BDL group, the pathways characterizing the primary functions of monocytes including antigen processing and presentation, FcγR mediated phagocytosis, and chemokine signaling, were significantly up-regulated in the VIR group. Our results reflected the outcome balanced between the subversion of monocyte transcriptome by HIV and the compensatory effects adapted by host cells. Furthermore, the altered pathways of antigen presentation and complement cascade highlighted that HIV manipulated adaptive immune response via innate immunity components at the interface of innate-adaptive immunity during disease progression. These data offered new comparative insights into the perturbed genetic networks of *ex vivo*-derived monocytes subverted by HIV during disease progression on therapy. In-depth functional studies on the regulation of these pathways and the corresponding core enrichment genes along with proteomic analysis may further confirm our findings and provide detailed molecular mechanisms underlying HIV-monocyte interaction.

## Methods

### Patient profiles and collection protocol

Five HIV+ patients on HAART who sustainably controlled HIV to below detection level (viral load < 40 copies of HIV RNA/ml), five HIV+ patients on HAART who consecutively experienced viremia, and four healthy HIV sero-negative controls were studied (Table 1). Patients with viral load < 1,000 were chosen to represent patients on HAART with virological failure because the previous study has demonstrated that the transcriptome profiling of these patients was more homogenous [23]. It has also been shown that monocyte transcriptomes from patients with viral load < 1,000 and those with higher viral load exhibited similar dysregulated pathways during disease progression. Patients in the VIR and BDL groups were on HAART for > 52 weeks. The BDL and VIR groups had a broad range of CD4+ T cell counts (100–796 cells/μl) as our objective was to find unbiased differences of gene expression profiling of monocytes between patients on HAART with sustained virus suppression and virological failure regardless of the degree of T cell decline. This grouping criterion by viral load has been successfully used by the previous studies [20,23,24]. These patients received two NRTIs (zidovudine, lamivudine, stavudine, emtricitabine, tenofovir) in association with one or two protease inhibitors (darunavir, ritonavir, indinavir, saquinavir, atazanavir). Ten patients were from the HIV clinic at Westmead Hospital and the four healthy controls were from the Australian Red Cross Blood Service in Sydney. This study was approved by the Sydney West Area Health Services Research Ethics Committee, and all blood samples were collected after individual informed written consent.

### Purification of CD14+ monocytes and RNA isolation

A single blood sample (10–20 ml in EDTA) was obtained from each patient. After separation of plasma, primary PBMCs were isolated immediately after obtaining blood samples by Ficoll-gradient centrifugation and purified. This aspect was strictly followed in our experiments because of previously described lower RNA yields and possible changes in gene expression profiles upon storage of blood [55]. CD14+ monocytes were then obtained by positive isolation with antibody-conjugated magnetic beads according to the manufacturer’s instructions (Miltenyi Biotech, Germany) with a purity > 98.6% as verified by flow cytometry. Binding of antibody to CD14 does not trigger signal transduction and a previous study has clearly demonstrated that CD14 positive selection does not alter cellular transcriptome by comparing gene expression profiles in parallel using either positive or negative selection [55]. Total RNA was isolated from purified cells using RNeasy Mini kit (Qiagen Pty Ltd., Clifton Hill, Victoria, Australia) with an integrated step of on-column DNase treatment.

### cRNA preparation, microarray hybridization and scanning

RNA quality was checked by Agilent Bioanalyzer and RNA Integrity Scores were higher than 9 for all the samples (Table 1). cRNA amplification and labeling with biotin were performed using Illumina TotalPrep RNA amplification kit (Ambion, Inc., Austin, USA) with 250 ng total RNA as input material. cRNA yields were quantified with Agilent Bioanalyzer and 750 ng cRNAs were hybridized to Illumina HumanHT-12 v3 Expression BeadChips (Illumina, Inc., San Diego, USA). Each chip contains 12 arrays and each array contains >48,000 gene transcripts, of which, 46,000 are derived from human genes in the National Center for Biotechnology Information (NCBI) Reference Sequence (RefSeq) and UniGene databases. All reagents and equipment used for hybridization were purchased from Illumina, Inc. According to the manufacturer’s protocol, cRNAs were hybridized to arrays for 16 hours at 58°C before being washed and stained with streptavidin-Cy3. Then the beadchips were centrifuged to dry and scanned on the Illumina BeadArray Reader confocal scanner. To minimize the batch effect, the microarray chips were all processed at the single site using the same platform with the identical setting of the parameters by the same experimenter. The microarray dataset has been submitted to GEO (Accession Number GSE52900).

### Analysis of differentially expressed genes

The quality of the entire data set was assessed by box plot and density plot of bead intensities, density plot of coefficient of variance, pairwise MAplot, pairwise plot with microarray correlation, cluster dendrogram, and non-metric multidimensional scaling using R/Bioconductor and the lumi package [56]. Based on the quality assessment, all 14 samples were deemed suitable for further analysis. Data normalization was performed using log2 transform and a robust spline normalization (RSN) implemented in the lumi package for R/Bioconductor [56,57]. Cluster analysis of gene expression profiling was carried out using dist and hclust functions from R stats package. Euclidean distance and complete linkage were used for distance metric and linkage criterion, respectively. To reduce false positives of differentially expressed genes, genes below detectable limit (based on a detection p value cut-off 0.01) were removed from the dataset. A linear model fit in conjunction with an empirical Bayes statistics was used to identify candidate DEGs [58]. P values were corrected for multiple testing using FDR adjustment implemented in lumi package. Pairwise comparisons for the 3 groups were carried out and candidate DEGs with fold change >2 and FDR <0.05 were identified for each of the comparisons. The list of the DEGs derived from the comparison of VIR versus BDL was uploaded to DAVID for the detection of the DEGs showing overlap with the genes in HIV interaction database at NCBI [59,60].

### Gene set enrichment analysis

GSEA was used for the comparison of our dataset with the published DEG lists from the previous studies (*in vivo and ex vivo* since 2002) [15,16,19,21,22,27-30], the investigation of global dysregulations of the biological pathways, and the promoter motif analysis. For the comparison with previous studies, 10 DEG lists were used from the studies on monocyte/MDM transcriptomes modulated by HIV (Table 4). For the pathway investigation, the gene sets were from MsigDB [32], catalog C2 functional sets, subcatalog KEGG pathways, which included 186 gene sets from pathway databases. For the promoter motif analysis, C3 motif gene sets which contained gene members sharing the same transcription factor binding site were used. This collection included 615 gene sets and each of them was annotated by a TRANSFAC record [33,61].

Instead of focusing on discrete DEGs, we analyzed the entire transcriptome data with GSEA to identify genes coordinately regulated in predefined gene sets from various biological pathways [32]. For each group comparison, GSEA was performed using the normalized data of entire 48,803 transcripts (GSEA version 2.07, Broad Institute http://www.broad.mit.edu/gsea). First, a ranked list was obtained by ranking all genes according to the correlation between their expression and the group distinction using the metric signal to noise ratio. Then the association between a given gene set and the group was measured by the non-parametric running sum statistic termed the enrichment score (ES), which was calculated by walking down the ranked list (increasing ES when encountering a gene in the given gene set and decreasing ES when encountering a gene not in the gene set). To estimate the statistical significance of the ES, a nominal p value was calculated by permuting the genes 1,000 times. To adjust for multiple hypothesis testing, the maximum ES was normalized to account for the gene set size (NES) and the false discovery rate (FDR) corresponding to each NES was also calculated. Along with the pathway enrichment results, the details report for each significant pathway was simultaneously generated. This report listed the details of each gene member in columns, one of which indicated whether this gene member was “core enrichment gene” or not. The core enrichment genes account for the enrichment signal of the pathway and the inspection of them can reveal a biologically important subset within the pathway [32].

### Real-time quantitative PCR

Ten genes and 14 pairs of group comparison were selected for validation based on the coverage of different levels and directions of fold change, different ranges of FDR values, and/or biological significance. Purified total cellular RNA was used for reverse transcription with oligo d (T) and Superscript III followed by RNase H treatment (Invitrogen Life Technologies). The cDNA was then subjected to qPCR in a 96-well format in triplicate reactions with defined primers and SYBR Green (Invitrogen Life Technologies). The qPCR reactions were carried out for the extended cohort consisting of 10 viremic patients, 10 BDLs, and 9 healthy controls (including all the original samples used in the microarray) using Mx3005P™ QPCR System (Stratagene). The mean expressions of the tested genes in each group were obtained and the housekeeping gene GAPDH was used as an internal control and the normalizer for all data. The fold change was calculated by the relative quantitation method 2^-(ddCt)^. Primer sequences for each transcript are available from the authors upon request.

## Competing interests

The authors declare that they have no competing interests.

## Authors’ contributions

JQW and TRS conducted the experiments, JQW analyzed the data and wrote the manuscript; MMS supervised the validation experiments; NKS and JQW designed the experiment; NKS supervised the work and assisted with writing the manuscript. All authors read and approved the final manuscript.

## Supplementary Material

Additional file 1 **Differentially expressed genes derived from the comparisons of VIR versus CTR and BDL versus CTR.** Description: The list of differentially expressed genes for the comparisons of VIR versus CTR and BDL versus CTR. Click here for file

Additional file 2 **Differentially expressed genes (VIR versus BDL) present in HIV interaction database.** Description: The list of differentially expressed genes (VIR versus BDL) present in HIV interaction database. Click here for file

Additional file 3 **Up-regulated pathways in the VIR group versus the BDL group from GSEA.** Description: The list of up-regulated pathways in the VIR group versus the BDL group from GSEA. Click here for file

Additional file 4 **Significantly altered gene sets in the VIR group versus the BDL group from promoter motif analysis by GSEA.** Description: The list of significantly altered gene sets in the VIR group versus the BDL group from promoter motif analysis by GSEA. Click here for file
